# Multi-Layer Artificial Neural Networks Based MPPT-Pitch Angle Control of a Tidal Stream Generator

**DOI:** 10.3390/s18051317

**Published:** 2018-04-24

**Authors:** Khaoula Ghefiri, Soufiene Bouallègue, Izaskun Garrido, Aitor J. Garrido, Joseph Haggège

**Affiliations:** 1 Laboratory of Research in Automatic Control—LA.R.A, National Engineering School of Tunis (ENIT), University of Tunis El Manar, BP 37, Le Belvédère, Tunis 1002, Tunisia; soufiene.bouallegue@issig.rnu.tn (S.B.); joseph.haggege@enit.rnu.tn (J.H.); 2 Automatic Control Group—ACG, Department of Automatic Control and Systems Engineering, Engineering School of Bilbao, University of the Basque Country, 48012 Bilbao, Spain; izaskun.garrido@ehu.es (I.G.); aitor.garrido@ehu.es (A.J.G.)

**Keywords:** artificial intelligence, artificial neural networks control, back-to-back converter, data processing, Doubly Fed Induction Generator (DFIG), Maximum Power Point Tracking (MPPT), pitch regulation, power control, Tidal Stream Generator (TSG)

## Abstract

Artificial intelligence technologies are widely investigated as a promising technique for tackling complex and ill-defined problems. In this context, artificial neural networks methodology has been considered as an effective tool to handle renewable energy systems. Thereby, the use of Tidal Stream Generator (TSG) systems aim to provide clean and reliable electrical power. However, the power captured from tidal currents is highly disturbed due to the swell effect and the periodicity of the tidal current phenomenon. In order to improve the quality of the generated power, this paper focuses on the power smoothing control. For this purpose, a novel Artificial Neural Network (ANN) is investigated and implemented to provide the proper rotational speed reference and the blade pitch angle. The ANN supervisor adequately switches the system in variable speed and power limitation modes. In order to recover the maximum power from the tides, a rotational speed control is applied to the rotor side converter following the Maximum Power Point Tracking (MPPT) generated from the ANN block. In case of strong tidal currents, a pitch angle control is set based on the ANN approach to keep the system operating within safe limits. Two study cases were performed to test the performance of the output power. Simulation results demonstrate that the implemented control strategies achieve a smoothed generated power in the case of swell disturbances.

## 1. Introduction

Along with many emerging ambient intelligence techniques, artificial intelligence is a useful tool that has been applied successfully in a broad range of multi-discipline problems [1]. The artificial neural network is one of those techniques that can deal with nonlinear problems in diverse applications in signal processing [2], pattern recognition [3] and extended to renewable energy converters [4]. The development of applications based on sensor devices has been widely used in power generation plants [5]. The main role of the sensors has been the continuous monitoring of the plant parameters until the past few years. Furthermore, some concerns regarding the plant security or the need of efficiency improvement make sensors an essential component within the control system. These devices play an important role in the control scheme, which represents a necessary tool for parameter measuring and monitoring that are then used as control input variables to the feedback loop [6]. The global warming, the emission of greenhouse and harmful gases, and dangerous climate impacts (storm, flood, etc.) are the various global problems [7]. In addition to these problems, the cost of production and high energy consumption and depletion of fossil energy deposits lead many countries’ policies to think seriously about their energy consumption [8]. In this context, several steps have been taken by several global constitutions to deal with economic and environmental issues. In this respect, the trends towards the study and exploitation of renewable energy have an inescapable necessity [9]. The world’s oceans provide a significant energy, which has recently been widely exploited [10]. In fact, the potential for electrical energy production from marine tidal currents is interesting [11]. Thus, the tidal currents are being considered as alternative and competitive to fossil fuel in the production of electrical energy [12]. In order to achieve the sustainable development, many countries tend to focus on the huge potential of ocean energy. European Policy has successfully taken these forms of energy into their future projections. By 2050, power generated by the ocean energy sector could avoid the equivalent of 276 m tonnes of CO_2_ emissions annually [13]. The worldwide potential for wave and tidal of renewable energy is approximately 337 GW [14].

The marine environment represents a source of energy, which could, theoretically, meet an important demand for power production; marine renewable energy converters are likely to play a significant part in a suite of technologies. This technology harvests the tidal current energy and extracts the kinetic energy from the flowing water. Environmental effects influence this form of energy. Thus, it is important that assessments of tidal current resources take into account more than the measured spring tidal currents [15]. In addition, it is not possible to consider the predictions based only on the nature of the natural flow [16]. Regarding the TSG systems, the swell effect is considered to be the most disturbing one for the tidal resource [17]. Tidal velocity can fluctuate due to the swell phenomenon and the induced changes in the tidal speed will lead to the disturbance of the TSG extractable power [18].

In the literature, several research works focused on the power output maximization of tidal stream converter systems [19]. The Maximum Power Point Tracking (MPPT) strategy is used to find the maximum power from tidal current and following the optimal regimes’ characteristics [20]. The control of the generated power in a variable speed operation is ensured by the use of power electronic converters. The rotor side converter control is used to keep the rotational speed of the generator at its optimal value and to minimize the core losses [21], while the grid side converter control aims to maintain the voltage of the DC-link and control the reactive power [22]. In variable speed mode, a control strategy of a Marine Current Turbine (MCT) system with a non-pitched blade angle has been implemented in [23]. The Doubly Fed Induction Generator-based MCT has been used within a rotational speed control scheme. The simulation results demonstrate that the implemented control strategy provides good tracking speed performances. Nevertheless, the active power shows some tracking errors. In case of power limitation mode, the pitch angle control is used in order to optimize the extracted power and shedding mechanical load. As discussed in [24], a comparison of the pitch and stall angle controls has been investigated. The study suggests that the stall-regulated systems are not able to keep a constant power output in the case of strong tidal velocity. The pitch angle control contributes to more efficient results regarding the energy yields. Research works focused on the pitch angle control have been discussed in [25]. According to [26], two control strategies are proposed to ensure the power limitation at high tidal speed. The speed and torque control strategies have been implemented. The obtained results show that both approaches are capable of limiting the produced power to the rated value at steady-state. However, the torque control strategy is more efficient than speed control strategy due to the fact that the generated power is more directly controlled during dynamic stages. In order to overcome the drawbacks of a fix-pitched angle control, a novel multilayer neural networks based-complementary control is investigated in this paper. This control combines both aforementioned strategies to provide a suitable smoothing switching tool for an efficient and robust tidal energy converter against the swell effect phenomenon.

This study aims to improve the performance and dynamic load assessment of the system under different operating conditions by adequately controlling the TSG transition under and above the tidal current speed threshold value. This novel complementary strategy focuses on the power smoothing control for the TSG system. The proposed control strategy is investigated using a multi-layer artificial neural networks strategy. This technique is used to provide the turbine rotational speed reference for which the maximum power is achieved and the pitch angular position in order to limit the generated power in the case of high tidal speeds. In power limitation mode, a comparative study of proportional and ANN-based controllers for the blade pitch angle of a Tidal Stream Turbine (TST) is discussed. ANN-based control aims to avoid frequent switching of the switch controller. In variable speed mode, a rotational speed control applied to the rotor side converter allows the system to follow the Maximum Power Point Tracking (MPPT) strategy. The principle of this control is to regulate the system so that, at each tidal speed, the turbine should track the rotational speed reference for which the maximum power is reached. The sensitivity of the proposed control strategies also have been evaluated in the case of turbulent tidal resources.

This paper is organized as follows; in Section 2, a background of the swell effect phenomenon and its influence on the power fluctuations is presented. After that, in Section 3, the TSG power plant is described and modeled. Section 4 implements the ANN-based control design of the MPPT-pitch angle strategy. Section 5 is devoted to the rotational speed control. In order to test the effectiveness of the proposed control approaches, two demonstrative study cases are presented and discussed in Section 6. Finally, concluding remarks are given in Section 7.

## 2. Fluctuation Aspects of Tidal Power

**Figure 1:**
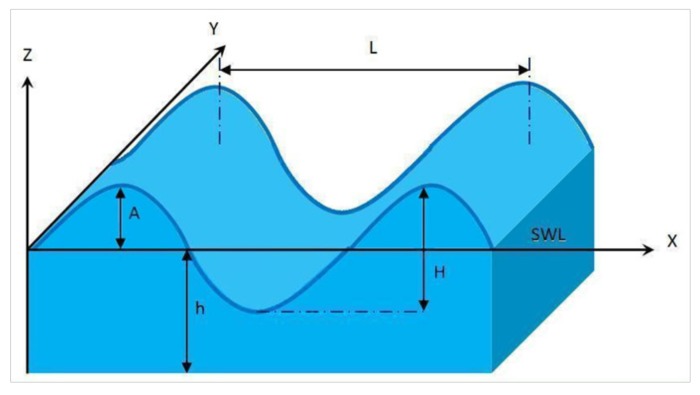
Swell characteristics.

In marine energy, there are two types of power disturbances: on a large time-scale that is related to the neap and spring tides, i.e., tidal velocity changes each 6 or 12 h and on a short-time scale, where the period is on the order of a few seconds [27]. In this sense, the spread of the swell underwater represents the main disturbance for the tidal speed as well as the major cause of the short-time fluctuations in the TSG framework. For that reason, the swell effect should be considered in the modeling of the tidal speed. It should be noted that the generated power from the TSG would fluctuate severely when there are turbulences in the tidal current speed. Figure 1 shows a scheme of the swell characteristics.

The center line marking the mean water level is called Still Water Level (SWL). It is used to measure the water depth *h* expressed in (m), which marks the distance from the seabed to the SWL. The portion of the wave profile with the highest elevation above the SWL is known as the wave crest and the part of the wave with the minimum depression is the wave trough. The distance between the wave trough and the wave crest is defined as the wave height *H* in (m). Furthermore, the distance from the SWL up to the crest or down to the trough is the amplitude of the wave denoted as *A* in (m). The wavelength *L*, expressed in (m), is the horizontal distance between the successive crests or successive troughs. The wavelength can also be defined as the horizontal distance between the successive points of equal amplitudes and phases.

The wavelength of a regular wave at any depth according to the linear theory can be defined as [28]:

(1) $$L\;=\;\frac{gT^{2}}{2\pi }\;\tanh\;\left(\frac{2\pi h}{L}\right),$$

where *g* is the gravity acceleration 9.81 ms${}^{-2}$ and *T* is the swell period expressed in seconds (s).

The swell effects on a tidal current velocity can be modeled using first order Stokes model [29]. The horizontal tidal speed (which is represented in the *x*-axis direction of Figure 1) can be determined using the following equation [30]:

(2) $$V_{x}\left(t\right)\;=\;\frac{\pi H}{T}\;\frac{\cosh\;(2\pi \left(\frac{z+h}{L}\right))}{\sinh(2\pi \frac{h}{L})}\;\cos\;2\pi \;\left(\;\frac{t}{T}-\frac{x}{L}\right),$$

where *x* and *z* are the horizontal and vertical point for the calculation.

According to Equation (2), only the sinusoidal swell is considered. However, from a real point of view, more than one frequency component should be taken into account to model the swell effects. Based on the JONSWAP spectrum and wave theories [31], each swell frequency component is determined. Assuming that $V_{rated}$ is the predicted rated tidal speed, which can be considered as a constant during a period less than one hour, the following equation is used to calculate the tidal velocity taking into account the swell effects [29]:

(3) $$V\left(t\right)\;=\;V_{rated}\;+\;\sum_{i}\frac{2\pi \;a_{i}}{T_{i}}\;\frac{\cosh\;(2\pi \left(\frac{z+h}{L_{i}}\right))}{\sinh(2\pi \frac{h}{L_{i}})}\;\;\cos\;2\pi \;\left(\;\frac{t}{T_{i}}-\frac{x}{L_{i}}+\varphi_{i}\right),$$

where $\varphi_{i}$ is the initial phase angle of each frequency component that is given randomly and $a_{i}$ represents the amplitude of each frequency component.

**Figure 2:**
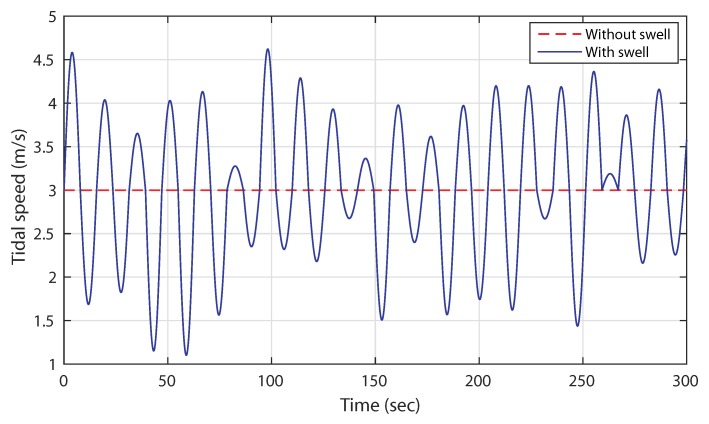
Tidal current speed profile with swell effect phenomenon.

Figure 2 shows the tidal current speed profile under the swell effect during 5 min. The rated tidal speed is considered at 3 m/s. It can be noted that the swell effect phenomenon can provoke large oscillations in the tidal speed for a given sea state. Consequently, these fluctuations induce high disturbances at the TSG extracted power.

## 3. Control Objectives and Modeling of the Tidal Stream Generator

### 3.1. Control Problem Statement

**Figure 3:**
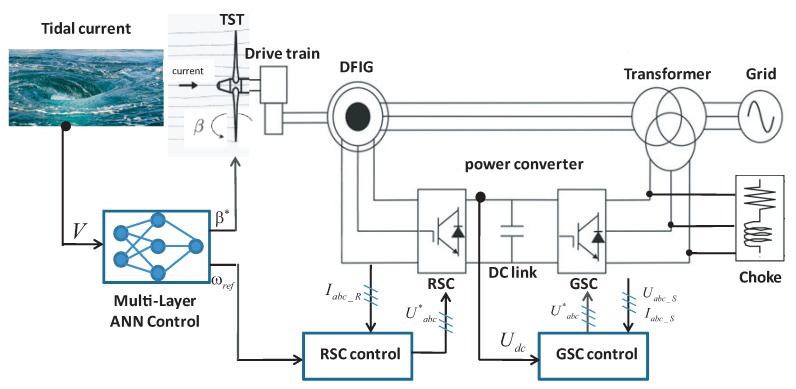
General scheme of a DFIG-based TST system and control description.

The TSG system consists of a Tidal Stream Turbine (TST) coupled to a Doubly Fed Induction Generator (DFIG) connected to the grid through the Back-to-Back power converter as shown in Figure 3. The turbine converts the kinetic energy from the tidal current speed to the rotation of the rotor shaft. The DFIG is coupled to the rotor shaft through the drive-train, which is adapted to the grid through the back-to-back power converters.

The control strategy describes how the tidal turbine is designed to approach the steady state of the ideal power curve. This amounts to adjusting the values of power and rotor speed at steady-state for each tidal speed in the range of the turbine operation. The control system may vary from one region of operation to another.

The ANN block provides two reference trajectories to the system: the suitable rotational speed reference following the MPPT strategy to maximize the extracted power; and the adequate angle of attack of the turbine blades to limit the power captured in the case of high tidal current speed. The control of the electrical part of the system is devoted to the DFIG focusing on the active and reactive powers’ control. This control approach is realized by the power electronics converters such as the Rotor Side Converter (RSC) and the Grid Side Converter (GSC). The RSC aims to maintain the rotational speed of the generator at an optimal value and minimizes the core losses while the GSC is used to maintain the voltage of the DC-link and controls the output reactive power.

### 3.2. Tidal Turbine Model

The hydrodynamic phenomenon of the tidal turbine is described by the following expression [32,33]:

(4) $$P_{t}=\frac{1}{2}C_{p}\left(\lambda ,\beta \right)\rho \pi R^{2}V^{3},$$

where $P_{t}$ represents the power captured from the tides in (W), *R* is the radius of the blades given in (m), ρ is the fluid density in (kg/m${}^{3}$) and *V* is the current speed in (m).

The tidal turbine does not use the total harnessed power from the tidal velocity due to Betz limit [34]. In addition, $C_{p}$ describes the power coefficient that depends on the pitch angle β in (deg) and the tip-speed ratio λ, expressed as follows [35]:

(5) $$\lambda =\frac{\omega_{t}R}{V},$$

where $\omega_{t}$ is the rotational speed of the rotor given in (rad/s).

The hydrodynamic turbine torque, expressed in (Nm), is governed by the following equation:

(6) $$T_{tst}=\frac{P_{t}}{\omega_{t}}.$$

### 3.3. Shaft Model

The tidal turbine is coupled to the DFIG via a drive train shaft. The two-mass model is used to describe the rotor shaft dynamics, which characterize the mechanical behavior of the turbine and the generator. The dynamic system is given as follows [36]:

(7) $$T_{tst}-T_{t}=2H_{t}\frac{d\omega_{t}}{dt},$$

(8) $$T_{t}=D_{sh}\left(\omega_{t}-\omega_{g}\right)+K_{sh}\int (\omega_{t}-\omega_{g})dt,$$

(9) $$T_{t}-T_{em}=2H_{g}\frac{d\omega_{g}}{dt},$$

where $T_{t}$ is the torque produced by the generator shaft in (Nm), $T_{em}$ is the electromagnetic torque in (Nm) and $\omega_{g}$ is the rotational speed of the generator in (rad/s). $K_{sh}$ is the stiffness coefficient in (Nm/rad), $D_{sh}$ is the damping coefficient in (Nms/rad), $H_{t}$ and $H_{g}$ are the inertia constants expressed in seconds for the tidal turbine and generator, respectively [37].

### 3.4. Generator Model

The variable speed operation of the DFIG-based TST has many advantages over other generator concepts. Their ability to generate power over a wide speed range in both sub- and super-synchronous modes makes them more attractive [19,38,39]. The dynamic model of the DFIG is defined in the synchronous rotating frame (*d-q*) using Park’s transformation [40]. The expressions of the stator voltages and flux, given in (V) and in (Wb) respectively, are defined as follows [41]:

(10) $$\left\{\begin{array}{c} U_{sd}=R_{s}I_{sd}+\frac{d\varphi_{sd}}{dt}-\omega_{s}\varphi_{sq},\\ U_{sq}=R_{s}I_{sq}+\frac{d\varphi_{sq}}{dt}-\omega_{s}\varphi_{sd}, \end{array}\right.$$

(11) $$\left\{\begin{array}{c} \varphi_{sd}=L_{s}I_{sd}+L_{m}I_{rd},\\ \varphi_{sq}=L_{s}I_{sq}+L_{m}I_{rq}. \end{array}\right.$$

The rotor voltages and flux are defined by the following equations [42]:

(12) $$\left\{\begin{array}{c} U_{rd}=R_{r}I_{rd}+\frac{d\varphi_{rd}}{dt}-\omega_{r}\varphi_{rq},\\ U_{rq}=R_{r}I_{rq}+\frac{d\varphi_{rq}}{dt}-\omega_{r}\varphi_{rd}, \end{array}\right.$$

(13) $$\left\{\begin{array}{c} \varphi_{rd}=L_{r}I_{rd}+L_{m}I_{sd},\\ \varphi_{rq}=L_{r}I_{rq}+L_{m}I_{sq}. \end{array}\right.$$

The electromagnetic torque of the generator is expressed in the *d-q* frame by Equation (14):

(14) $$T_{em}=\frac{3}{2}pL_{m}\left(I_{sq}I_{rd}-I_{sd}I_{rq}\right),$$

where $I_{sd}$, $I_{sq}$ are the stator currents defined in *d-q* synchronous frame in (A), $I_{rd}$, $I_{rq}$ are the rotor currents defined in *d-q* axis in (A), $R_{s}$ and $R_{r}$ are the stator and rotor resistances in (Ω), $\omega_{s}$ and $\omega_{r}$ are the stator and rotor pulsations in (rad/s), $L_{s}$ and $L_{r}$ are the stator and rotor inductances in (H), $L_{m}$ is the magnetizing inductance in (H) and *p* is the pole pair number.

### 3.5. Power Converters Model

The use of the power electronics converter is the key to connect the system to the grid via the DC-link capacitor [40,41,42,43,44]. The AC-DC-AC converter is formed by the GSC connected to the grid and the RSC connected to the DFIG as depicted in Figure 3. The GSC is intended to keep the DC-link voltage constant regardless of the magnitude and direction of the rotor power using a vector control strategy [45]. However, the role of RSC is improving the operation of the DFIG providing maximum power production using a vector control strategy [46].

The equations of the active and reactive powers of the system, expressed in (W) and (VAR) respectively, are given as follows [47]:

(15) $$P_{g}=\frac{3}{2}\left(U_{dg}I_{dg}-U_{qg}I_{qg}\right),$$

(16) $$Q_{g}=\frac{3}{2}\left(U_{qg}I_{dg}-U_{dg}I_{qg}\right),$$

where $U_{dg}$, $U_{qg}$ in (V) and $I_{dg}$, $I_{qg}$ in (A) are the grid voltages and currents defined in the *d-q* reference frame, respectively.

In order to achieve the voltage oriented control, the *d*-axis of the synchronous frame and the grid voltage vector are aligned, i.e., $U_{dg}=U_{g}$ and $U_{qg}=0$. Hence, the equations of the active and reactive powers can be rewritten as follows [47]:

(17) $$P_{g}=\frac{3}{2}U_{g}I_{dg},$$

(18) $$Q_{g}=-\frac{3}{2}U_{g}I_{qg}.$$

The expression between the power stored in the DC-link and the power transferred to the grid is defined by Equation (19) [48]:

(19) $$P_{g}=\frac{3}{2}U_{g}I_{dg}=U_{dc}I_{dc},$$

where $U_{dc}$ in (V) is the DC bus voltage and $I_{dc}$ in (A) is the current accross the DC-link.

## 4. Artificial Neural Networks Based-Planning Control Trajectories

An Artificial Neural Network can efficiently approximate and interpolate multivariate data that might otherwise require huge databases [49]. Such a technique is well accepted for nonlinear statistical fitting applications [50,51]. The nonlinearity aspect of the tidal stream turbine along with the integration of the DFIG and the connection to the grid through the back-to-back power converter makes this system more complex. In this context, a multi-layer ANN is proposed as a promising solution to deal with the nonlinearity of the system from tides to the grid. The implemented ANN block aims to provide two reference trajectories. On one hand, the turbine rotational speed reference for which the maximum power is achieved. On the other hand, the pitch angular position in order to limit the generated power in the case of high tidal speeds.

### 4.1. Multi-Layer ANN Control Design

**Figure 4:**
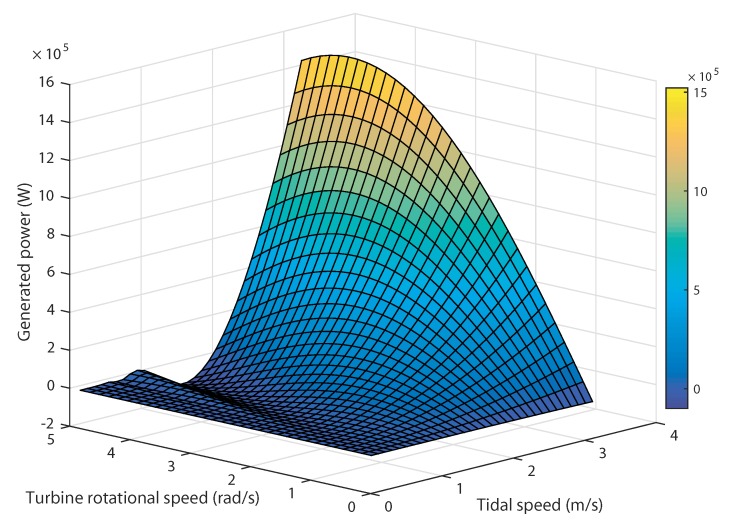
Generated power versus rotational speed for different tidal speeds.

In a TSG system, the turbine exhibits an optimal performance at a specific rotational speed. For each tidal speed, there is a certain rotational speed at which the harnessed power reaches its maximum value [52]. Figure 4 shows the variation of the generated power versus the rotational speed for different tidal currents.

All these maxima define what is known in the literature as the Optimal Regimes Characteristic (ORC) [53] . According to the turbine under study, the maximum power coefficient is $C_{p\max}=0.44$ corresponding to the optimal tip-speed ratio $\lambda_{opt}=6.34$. The maximum extractable power is $P_{n}$ = 1.5 MW at the rated tidal speed $V_{n}$ = 3.2 m/s. In this variable speed mode, the generated power varies as the tidal current speeds are changing for certain rotational speed for a fixed pitch angle assumed null. From this simulation, it can be noted that, for each specific tidal speed input, the tidal turbine can harness the maximum power for a certain rotational speed.

**Figure 5:**
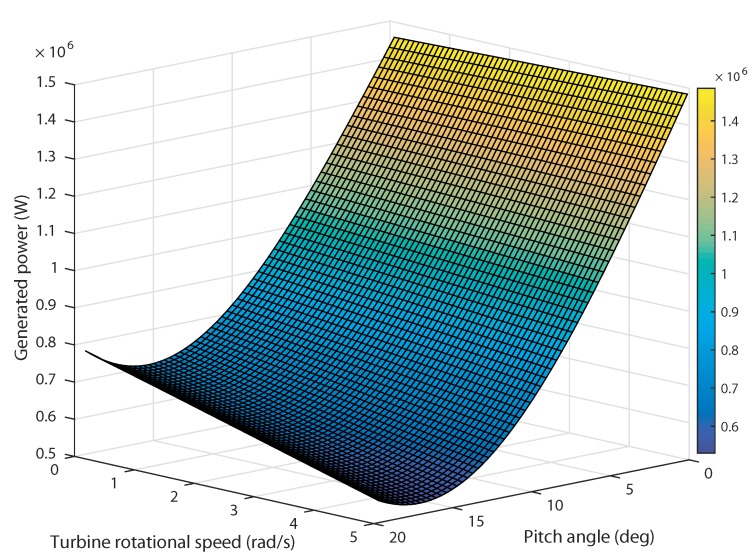
Generated power versus rotational speed for different pitch angles at rated tidal speed.

In the case of high tidal speeds and strong waves, the pitch angle control is used to limit the harnessed power by adequately rotating the blades at the suitable angular position. As depicted in Figure 5, the generated power varies with the rotational speed for different values of the pitch angle at the rated tidal speed $V_{n}$. The maximum power is reached at $\beta =0^{\circ }$. It should be noted that as the pitch angle augments the generated output power decreases for a specific rotational speed. This study shows the effect of the pitch angle control to keep the system operating within safe specified limits.

**Figure 6:**
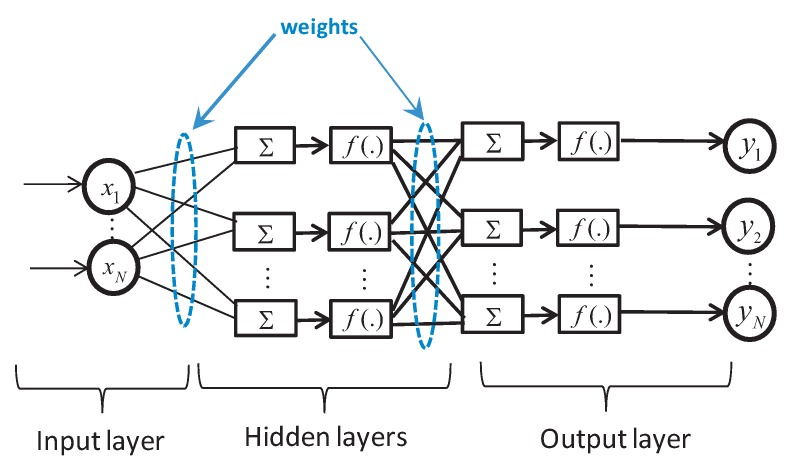
Layout of a multilayer ANN.

Based on the characteristic curves of the studied TST, the feed-forward ANN is designed to generate the MPPT reference to the RSC’s controller and the desired pitch angle to the pitch actuator. The architecture of a multi-layer ANN, in the general case, consists of the input, hidden and output layers as shown in Figure 6. The information flowing from the input to the output neurons is assured by the neurons of the hidden layers with the appropriate weights and activation functions.

In order to design the ANN block, we have to set the appropriate input and output data of the TST system. The ANN block is trained in the way that it can generate the suitable rotational speed reference following the MPPT strategy and fixing the pitch angle null. When the tidal turbine reaches the maximum power, the system should switch to assure the required pitch angle for each input data to prevent the overload of the turbine. The implemented feed-forward network is composed of one input layer with one neuron, which represents the tidal speed variation and one output layer with two neurons. Due to the complexity of the multi-physic system which fully integrates the hydrodynamic loads, turbine, induction generator and the back-to-back converter models, the authors opted for using the trial-and-error rule based on forward approach procedure [54]. This starts with undersized number of hidden neurons and adds the number of neurons. After that, train and test the neural network. Then, the number of hidden neurons is increased and the above procedure repeated until the training and testing results are improved. This method is based on a statistical analysis by satisfying as performance criteria the best mean squared error achieved. During simulation, several values of neurons of the hidden layer were tested in order to obtain the smallest Mean Squared Error (MSE) to achieve the best validation performance. The activation function used in the hidden layer is with the hyperbolic tangent type, whereas that corresponding to the output neuron was chosen linear.

After adjusting the structure of the neuro-controllers, the Levenberg–Marquardt (LM) algorithm is adopted as a learning method for training the ANN [55]. The LM algorithm is a variation of Newton’s method that was designed for minimizing functions that are sums of squares of other nonlinear functions [56]. The algorithm should adjust the network parameters in order to minimize the performance index as follows [57]:

(20) $$\Delta \;X=\;{\left[\nabla^{2}F\left(X\right)\right]}^{-1}\;\nabla F\left(X\right),$$

where $\nabla^{2}F\left(X\right)$ is the Hessian matrix and ∇F(X) is the gradient.

Considering F(X) as the performance index, which is defined as follows:

(21) $$F\left(X\right)=\;\sum_{i=1}^{N}e_{i}^{2}\left(X\right)=e^{T}\left(X\right)\;e\left(X\right).$$

Its gradient may be rewritten as:

(22) $$\nabla F\left(X\right)=2\;J^{T}\left(X\right)\;e\left(X\right),$$

where J(X) is the Jacobian matrix.

The LM algorithm implementation requires the calculation of the Jacobian matrix, with a size proportional to the number of training patterns as follows [58]:

(23) $$J\left(X\right)=\;\left(\begin{array}{ccc} \frac{\partial e_{1}\left(X\right)}{\partial X_{1}} & \frac{\partial e_{1}\left(X\right)}{\partial X_{2}} & \cdots \;\;\;\frac{\partial e_{1}\left(X\right)}{\partial X_{n}}\\ \begin{array}{c} \frac{\partial e_{2}\left(X\right)}{\partial X_{1}}\\ \;\;\;\;\;\vdots \end{array} & \begin{array}{c} \frac{\partial e_{2}\left(X\right)}{\partial X_{2}}\\ \;\;\;\;\vdots \end{array} & \begin{array}{c} \cdots \;\;\;\frac{\partial e_{2}\left(X\right)}{\partial X_{n}}\\ \ddots \;\;\;\;\;\;\;\vdots \end{array}\\ \frac{\partial e_{N}\left(X\right)}{\partial X_{1}} & \frac{\partial e_{N}\left(X\right)}{\partial X_{2}} & \cdots \;\;\;\frac{\partial e_{N}\left(X\right)}{\partial X_{n}} \end{array}\right).$$

Then, the Hessian matrix can be expressed in the following form:

(24) $$\nabla^{2}F\left(X\right)=2\;J^{T}\left(X\right)\;J\left(X\right)+2S\left(X\right),$$

where:

(25) $$S\left(X\right)=\sum_{i=1}^{N}e_{i}\left(X\right)\nabla^{2}e_{i}\left(X\right).$$

In order to select the adequate number of neurons of the hidden layer, an empirical comparative study is performed through different tests. The evaluation of the performance of the used LM algorithm was established taking into account the number of the epochs and the mean squared error found. Table A1 of Appendix A shows the performance of the training process for different numbers of neurons in the hidden layer.

Training tests show that, by increasing the number of neurons in the hidden layer from 2 to 11, the LM algorithm reaches a lower optimization stopping criteria for a higher number of epochs. The MSE increases for $h_{i}$ higher than 10, which indicates that the network can be overtrained using those patterns.

### 4.2. Training Performance of ANN-Based Controller

**Figure 7:**
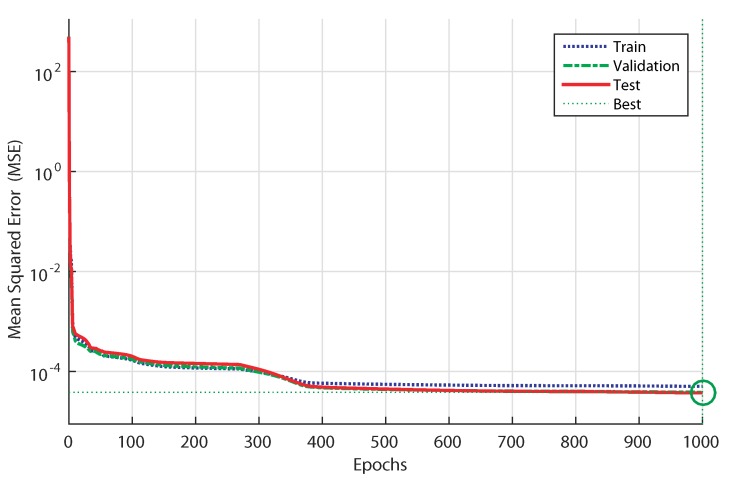
Performances of the multi-layer ANN training.

The best validation performance is 38,614·$10^{-5}$ at 1000 epochs which corresponds to $h_{i}=10$. Figure 7 indicates the iteration at which the validation performance reached a minimum. This feature shows that the training data indicates a good fit.

**Figure 8:**
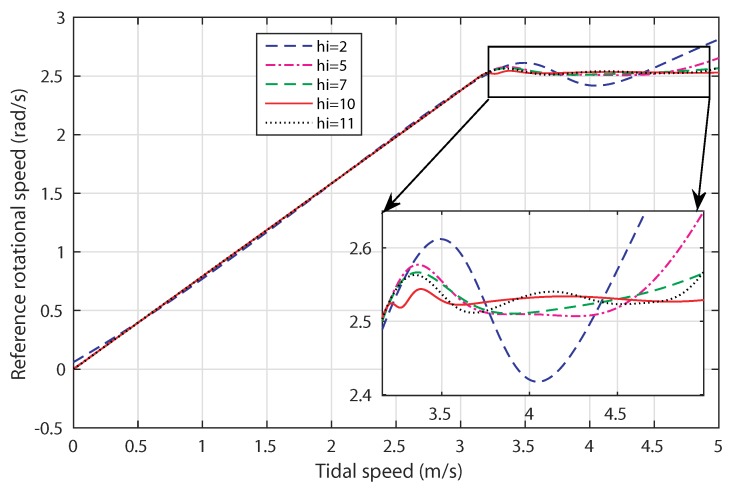
Response of the ANN-based MPPT control.

Figure 8 shows the response of the MPPT based-ANN control strategy by changing $h_{i}$ for the tidal speed ranging from 0 to 5 m/s. When the tidal speed varies over the range $\left[0\;\;,\;\;3.2\;m/s\right]$, the rotational speed reference is adjusted to track the optimal reference for which the power coefficient is at its maximum value $\left(C_{p\max}=0.44\right)$. Once the tidal speed reaches the tolerable value, which is 3.2 m/s, the speed reference should maintain the nominal value $\omega_{n}$ = 2.53 rad/s. Note that all the generated references ensure the MPPT in the variable speed operation mode, whereas, in power limitation mode, the trajectory of the rotational speed, which corresponds to $h_{i}=10$, is the most stable response and is able to keep $\omega_{ref}$ around $\omega_{n}$.

**Figure 9:**
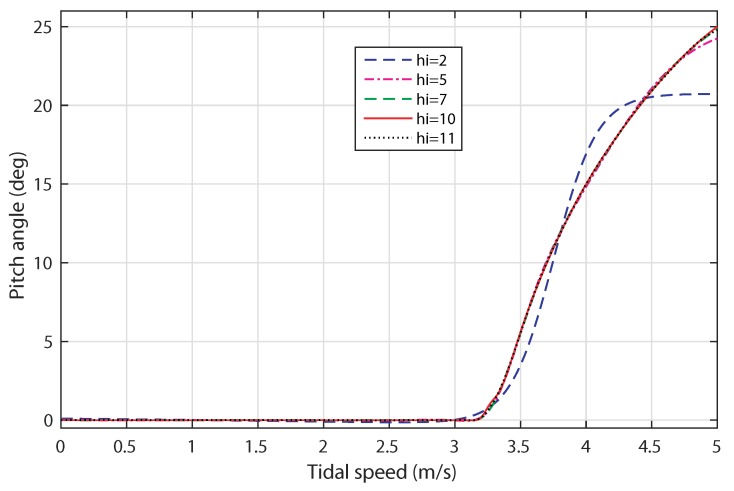
Response of the ANN-based pitch angle control.

Figure 9 shows the response of the pitch angle control based-ANN by varying the tidal speed from 0 to 5 m/s. When the tidal speed varies over the range $\left[0\;\;,\;\;3.2\;m/s\right]$, the pitch angle response is null for all the tested $h_{i}$. As the tidal speed reaches 3.2 m/s, the value of the pitch angle increases accordingly with the variation of the input.

## 5. Rotational Speed Control of TSG System

### 5.1. Rotor Side Converter Control

**Figure 10:**
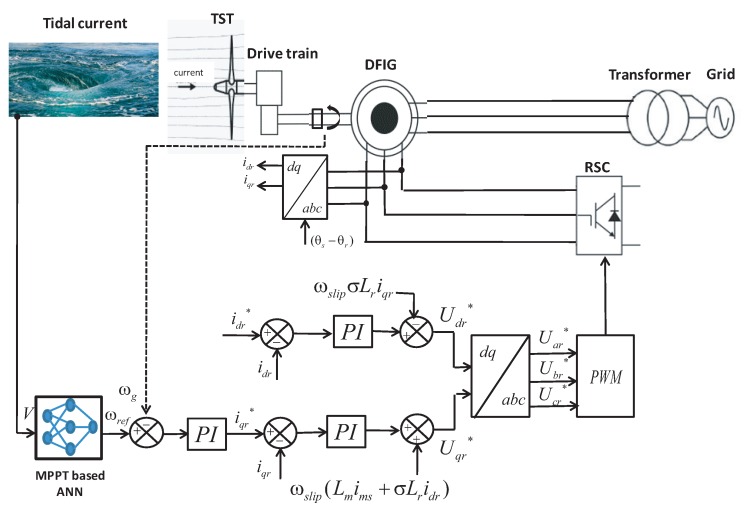
Stator flux oriented control strategy for the RSC.

For the RSC, the stator flux control strategy is applied as depicted in Figure 10. The needed variables in this control scheme are the stator and rotor currents, the stator voltage and the rotor speed. The control design structure consists of one outer loop for the rotational speed and two inner loops for currents’ regulation.

The reference speed $\omega_{ref}$ is generated from the implemented ANN block as discussed previously in Section 4 according to the MPPT approach. The ANN output is used for the speed control loop, which defines a reference for the *q*-axis rotor current $i_{qr}^{*}$. The *d*-axis current reference $i_{dr}^{*}$ is set to zero, whereas the inner current control loop determines the *d-q* rotor voltage reference. The expressions that link between the rotor voltages in (V) and currents in (A) are defined by Equation (26) as given in [40]:

(26) $$\left\{\begin{array}{c} U_{dr}=R_{r}i_{dr}+\sigma L_{r}\frac{di_{dr}}{dt},\\ U_{qr}=R_{r}i_{qr}+\sigma L_{r}\frac{di_{qr}}{dt}, \end{array}\right.$$

where σ is the leakage factor.

Furthermore, decoupling terms are added to the equations of $U_{dr}^{*}$ and $U_{qr}^{*}$ that will improve the transient response of the system [59]. Thus, the rotor voltage references are given as follows:

(27) $$\left\{\begin{array}{c} U_{dr}^{*}=-\omega_{slip}\sigma L_{r}i_{qr}+\left(K_{Pi}e_{d}+K_{Ii}\int e_{d}\;dt\right),\\ U_{qr}^{*}=\omega_{slip}\left(L_{m}i_{m}+\sigma L_{r}i_{dr}\right)+\left(K_{Pi}e_{d}+K_{Ii}\int e_{d}\;dt\right), \end{array}\right.$$

where $\omega_{slip}$ is the slip angular frequency in (rad/s) and $i_{m}$ is the stator magnetizing current assumed as constant. $K_{Pi}$ and $K_{Ii}$ denote the gains of PI controllers.

The PI controllers design is performed using the Ziegler–Nichols method [60]. Then, using the robust response time algorithm, further refinement on the initial value of the PI gains were applied [61]. The rotor voltage references are transformed to the three-phase abc stationary frame to be applied to the RSC via the Pulse Width Modulation (PWM) block.

### 5.2. Grid Side Converter Control

**Figure 11:**
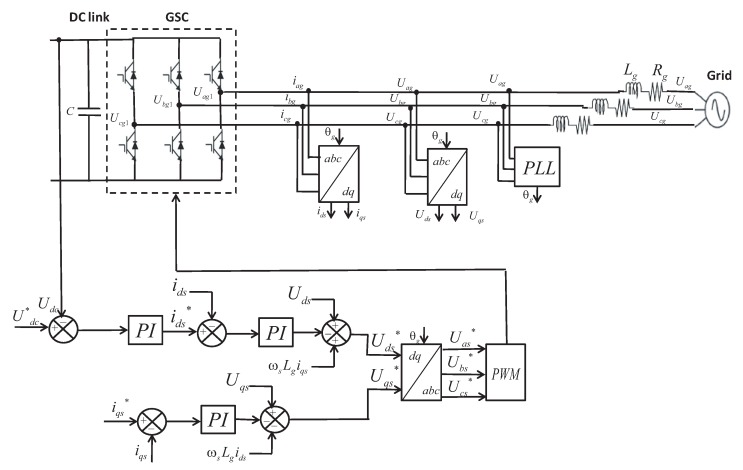
Voltage oriented control strategy for the GSC.

The GSC is controlled by the voltage oriented control scheme as depicted in Figure 11. The control strategy consists of two series of PI controllers within a cascade configuration. The implemented scheme controls the DC-link voltage $U_{dc}$ and the reactive power $Q_{g}$. The Phase Locked Loop (PLL) block is used to recover the phase of the input signal, which is $\theta_{g}$. The direct and quadrature components of the currents in (A) and voltages in (V) are obtained using Park’s transformation.

The grid voltages in (V) are defined in the *d-q* reference frame as follows:

(28) $$\left\{\begin{array}{c} U_{gd}=i_{ds}R_{g}+L_{g}\frac{di_{ds}}{dt}-\omega_{s}L_{g}i_{qs}+U_{gd1},\\ U_{gq}=i_{qs}R_{g}+L_{g}\frac{di_{qs}}{dt}-\omega_{s}L_{g}i_{ds}+U_{gq1}, \end{array}\right.$$

where $R_{g}$ in (Ω) and $L_{g}$ in (H) are the grid coupling resistance and inductance respectively, $U_{gd1}$ and $U_{gq1}$ in (V) are the two phase converter terminal voltages.

According to Equations (17) and (18), the active and reactive powers are controlled via the *d*-axis and *q*-axis current, respectively. The current control loops are identical and generate the grid voltage references $U_{ds}^{*}$ and $U_{qs}^{*}$ defined by Equation (29). Then, compensator terms and feed-forward voltages are added to the control signals in order to enhance the transient response of the system [62]:

(29) $$\left\{\begin{array}{c} U_{gd}^{*}=U_{gd}+\Omega_{g}L_{g}i_{q}-\left(K_{Pi}e_{d}+K_{Ii}\int e_{d}\;dt\right),\\ U_{gq}^{*}=U_{gq}-\Omega_{g}L_{g}i_{d}-\left(K_{Pi}e_{q}+K_{Ii}\int e_{q}\;dt\right). \end{array}\right.$$

The outer voltage loop is designed to control the DC voltage $U_{dc}$ and keep it constant. The two inner current loops are intended to regulate the *d*-axis and *q*-axis currents $i_{ds}$ and $i_{qs}$. The quadrature current component $i_{qs}$ is used to regulate the reactive power. During the normal operation, the converter will transfer all the generated TST active power to the grid. Thus, the *q*-axis current reference is assuming zero. Similarly to the RSC case, the design of the PI controllers is carried out using the experimental Ziegler–Nichols method. Finally, the reference voltages transformed to the three phase abc frame are then used to generate all PWM signals for the GSC block.

## 6. Validation Tests and Discussion

**Figure 12:**
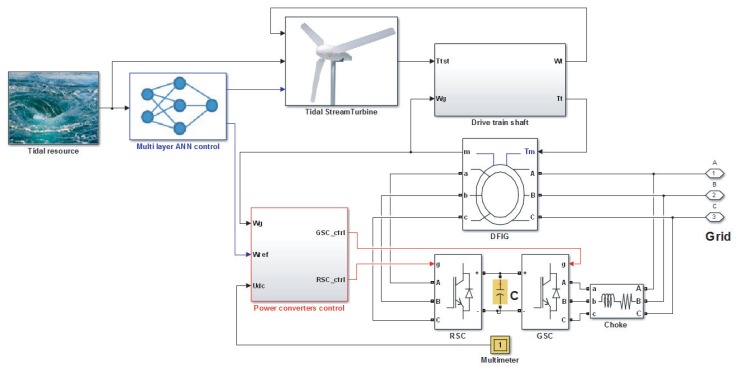
Numerical implementation of the TSG model.

In this section, two study cases are presented to investigate the effectivness of the proposed control strategies in order to improve the generated power output and to test the robustness of the ANN control against swell effect disturbances. Simulations have been carried out to validate the proposed ANN-based control using the model described in Section 3 and implemented as shown in Figure 12. For all performed simulations, the used model parameters are listed in Table A2 of Appendix A.

### 6.1. Comparative Study between the Switching and ANN-Based Controls

In order to test the effectiveness of the proposed control strategies, this study case is set to compare the ANN-based control scheme with a switching control investigated in previous research work [32] for the same TSG system.

**Figure 13:**
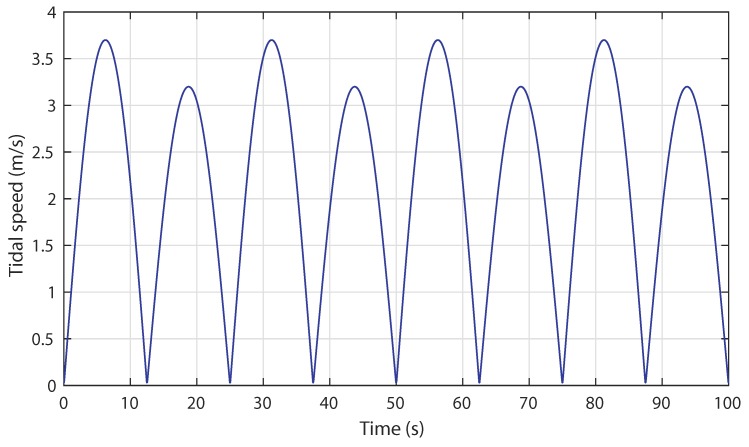
Scenario 1: regular tidal speed input.

The scenario considered for the tidal current speed takes the shape of a semidiurnal spring and neap tides. The tidal speed input is illustrated in Figure 13. It can be seen that the maximum pic of the spring and neap tides achieve the value 3.6 m/s and 3.2 m/s, respectively.

**Figure 14:**
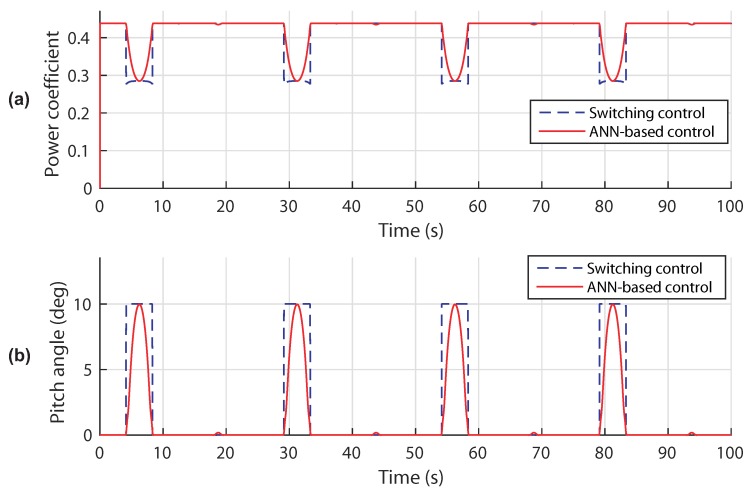
Control performances in scenario 1: (**a**) power coefficient response; (**b**) pitch angle response.

Figure 14 illustrates the comparison between the responses of the power coefficient and the pitch angle with a conventional switch controller and ANN-based strategy. It is obvious that ANN control successfully adjusts the blade pitch angle as well as the power coefficient with the change of the tidal speed. In contrast, the switching control does not adapt well to all input changes. The power coefficient is maintained at its maximum value 0.438 in variable speed mode and decreases at high tidal velocity. Consequently, the angular position of the turbine blades is kept null in the variable speed mode and increases in the power limitation mode. It is noted that the generated signals are time varying, which makes the proportional feedback inefficient. By using a multi-layer ANN-based controller in the power limitation mode, the tracking performances of the adequate pitch angle of the blades at each tidal speed are clearly improved.

**Figure 15:**
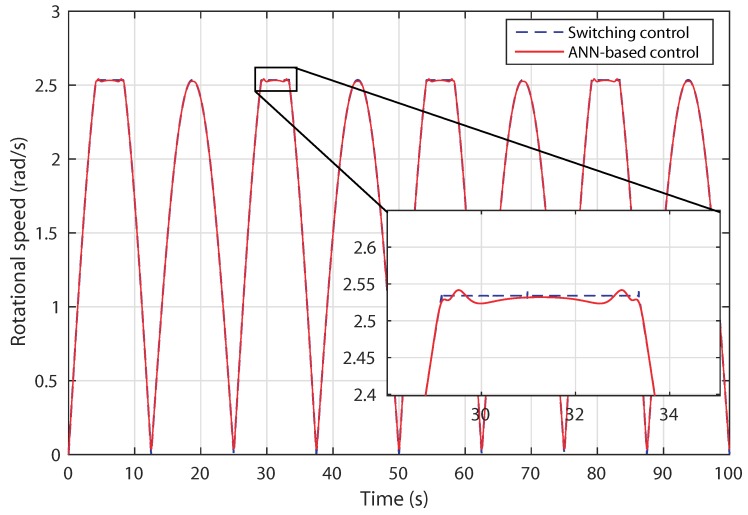
Scenario 1: turbine rotational speed variation.

The rotor speed increases according to the flow speed input and it is kept constant below the threshold value as depicted in Figure 15. On one hand, when the tidal speed is below the rated value (3.2 m/s), both control approaches lead to a good tracking of the rotational speed. On the other hand, when tidal current is higher than the rated value, the ANN-based controller shows an improved tracking of the rotational speed.

**Figure 16:**
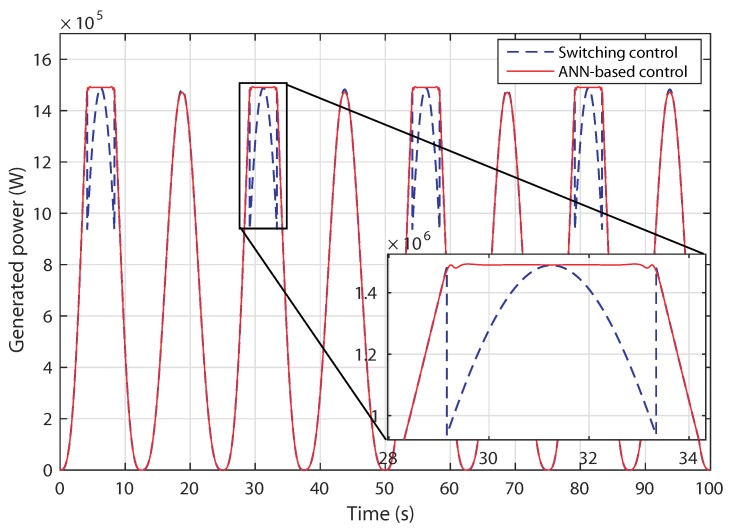
Scenario 1: generated active power variation.

The resulting generated power variation is shown in Figure 16. The tidal turbine is able to maximize the generated power below nominal tidal velocity and then is kept at the nominal power. By using the proposed ANN control, it is obvious that the peak of change during the transition between two modes is eliminated. This leads to a power generation improvement.

### 6.2. Robustness of the ANN-Based Control against Swell Effects

**Figure 17:**
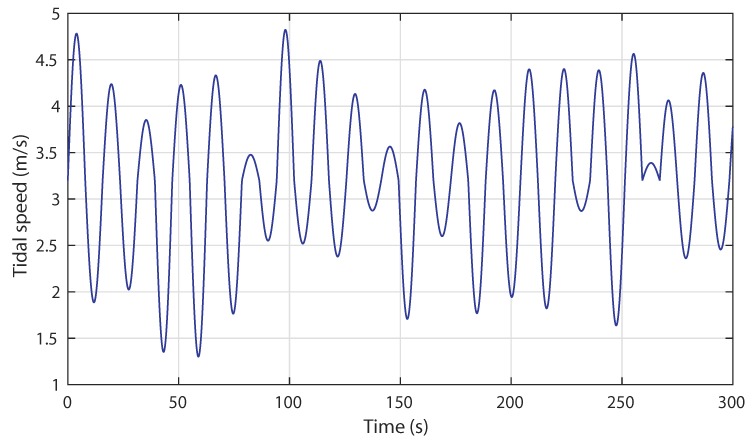
Scenario 2: Turbulent tidal speed input.

In this subsection, the proposed ANN control strategy is analyzed regarding the swell effect disturbances. Based on the model previously developed in Section 2, a disturbed tidal resource with a minimum speed of 1.3 m/s and a maximum speed of 4.8 m/s is set. The turbulent resource characteristic is given in Figure 17.

**Figure 18:**
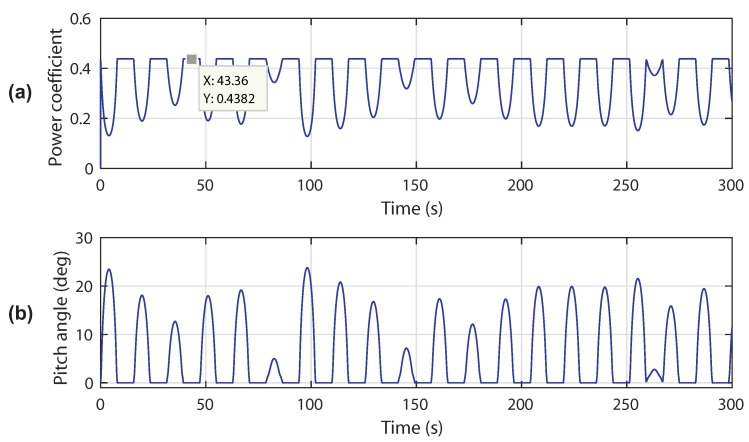
Control performances in scenario 2: (**a**) power coefficient response; (**b**) pitch angle response.

Figure 18 illustrates the power coefficient and pitch angle responses. In this experiment, it can be seen that the power coefficient response is kept around its optimum value $C_{p}=0.4382$ and adequately varies in the case of high tidal velocity. The resulting pitch angle increases accordingly with the tidal speed variation.

**Figure 19:**
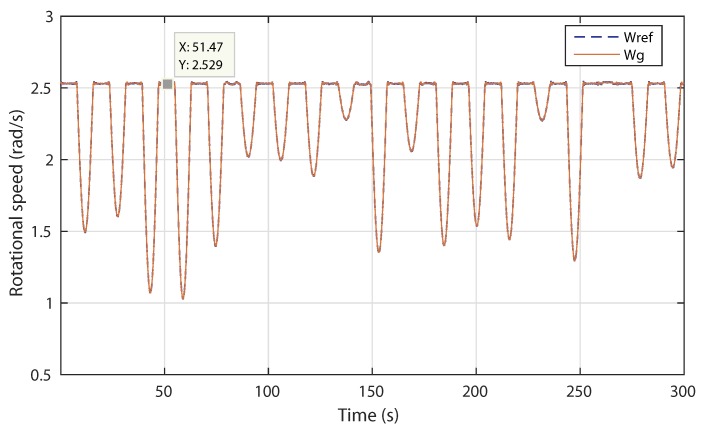
Scenario 2: Generated reference and actual rotational speed.

Figure 19 illustrates the variation of the rotational speed and the reference obtained with the proposed MPPT strategy. The control system performs well because the rotational speed shows a good tracking performance of the reference signal.

**Figure 20:**
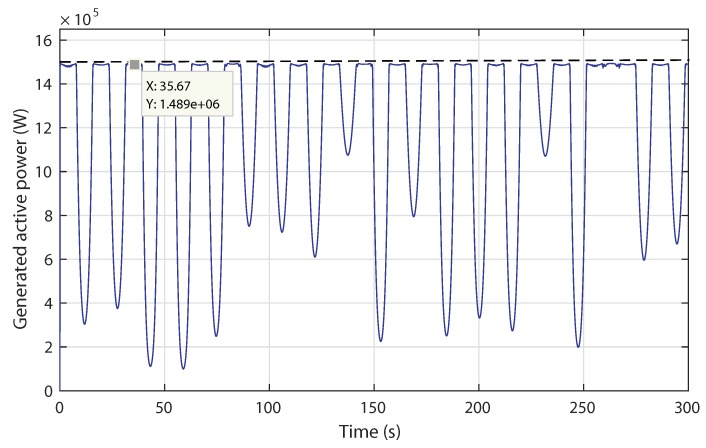
Scenario 2: Generated active power response.

The produced power under swell effect is shown in Figure 20. The proposed control is able to limit the power generated at $P_{n}$ when the tidal current speed exceeds $V_{n}$. The ANN complementary control ensures a smoothing transition between the variable speed mode and the power limitation mode. Simulation results show that the novel control strategy is excellent in terms of speed tracking and power regulation.

## 7. Conclusions

In this paper, a DFIG-based TST system has been modeled and controlled. Two control strategies have been designed and implemented to deal with the power fluctuations due to the swell effect phenomenon. The first control approach is elaborated around a novel complementary ANN-based MPPT pitch angle control strategy. On one hand, the MPPT approach aims to track the suitable rotational speed in order to achieve the maximum power generation. On the other hand, the pitch angle controller adequately regulates the angular position of the turbine blades in order to limit the extracted power for high tidal current speeds. The second control strategy is devoted to the rotational speed regulation by means of the rotor side and grid side converters. A stator flux-oriented control scheme was applied to the RSC and a voltage-oriented control was used for the GSC. The implemented control scheme aims to alternate between both operation modes. That is, the TSG is regulated so as to smoothly pass from the variable speed mode to the power limitation mode in order to optimize the generated output power and to ensure the protection of the system from overloading.

To test the effectiveness of the complementary ANN controller, a scenario is proposed with a variable spring and neap tidal current speed. This control strategy was introduced to solve the problems of the switching control. The obtained results prove that the implemented control has an advantage of eliminating the pic at the output signal and that the response time between the variable speed and power limitation modes is reduced. This proposed ANN-based pitch angle control makes the output signal smoother and eliminates signal discontinuity, which is a drawback when applying the conventional proportional controller. By using the proposed novel control strategy, the transition between controls is successfully smoothed given an input variable signal with the shape of the real tidal speed.

A second scenario was carried out to test the robustness of the ANN control strategy against swell effect disturbances. Results prove that the controller successfully overcomes these fluctuations, enabling the TSG system to extract the maximum power. In addition, this control strategy provides an excellent performance and improves the power generation regarding a fluctuated tidal resource.

## Appendix A

**Table A1:** Levenberg–Marquardt training performances.

Neurons Number in Hidden Layer	Epochs	MSE
	13	0.032676
	18	0.049599
2	21	0.036628
	35	0.03845
	41	0.029477
	58	0.034446
	70	0.0004752
	153	0.00038727
5	187	0.00053197
	225	0.00049101
	326	0.000532
	714	0.0001963
	69	0.00016313
	138	0.00019381
7	222	0.0002327
	653	0.00017829
	751	0.00014904
	1000	0.00017071
	118	0.00012838
	361	$9.1106\times 10^{-5}$
10	649	$8.7021\times 10^{-5}$
	720	$6.2352\times 10^{-5}$
	955	$4.3441\times 10^{-5}$
	1000	$3.8614\times 10^{-5}$
	115	0.00019116
	157	0.00014699
11	241	0.00011463
	623	$9.5236\times 10^{-5}$
	904	$8.9541\times 10^{-5}$
	1000	$5.3738\times 10^{-5}$

**Table A2:** TSG system parameters.

Turbine	Drive-Train	DFIG	Converter
ρ = 1027kg/m${}^{3}$	$H_{t}$ = 3 s	$P_{n}$ = 1.5MW	$V_{dc}$ = 1150V
R = 8m	$H_{g}$ = 0.5 s	$U_{rms}$ = 690V	C = 0.01F
$C_{p\max}=0.44$	$K_{sh}=2\times 10^{6}$ Nm/rad	$f_{req}$ = 50Hz	
$\lambda_{opt}=6.96$	$D_{sh}=3.5\;\times 10^{5}$ Nms/rad	$R_{s}=2.63\;m\;\Omega$	
$V_{n}$ = 3.2m/s		$R_{r}=2.63\;m\;\Omega$	Choke
		$L_{s}=0.168\;\mathrm{mH}$	$R_{g}=0.595\;m\Omega$
		$L_{r}=0.133\;\mathrm{mH}$	$L_{g}=0.157\;\mathrm{mH}$
		$L_{m}=5.474\;\mathrm{mH}$	
		p=2	
